# Automated imaging-based abdominal organ segmentation and quality control in 20,000 participants of the UK Biobank and German National Cohort Studies

**DOI:** 10.1038/s41598-022-23632-9

**Published:** 2022-11-04

**Authors:** Turkay Kart, Marc Fischer, Stefan Winzeck, Ben Glocker, Wenjia Bai, Robin Bülow, Carina Emmel, Lena Friedrich, Hans-Ulrich Kauczor, Thomas Keil, Thomas Kröncke, Philipp Mayer, Thoralf Niendorf, Annette Peters, Tobias Pischon, Benedikt M. Schaarschmidt, Börge Schmidt, Matthias B. Schulze, Lale Umutle, Henry Völzke, Thomas Küstner, Fabian Bamberg, Bernhard Schölkopf, Daniel Rueckert, Sergios Gatidis

**Affiliations:** 1 Biomedical Image Analysis Group, Department of Computing, Imperial College London, London, UK; 2 Institute of Signal Processing and System Theory, University of Stuttgart, Stuttgart, Germany; 3 Department of Brain Sciences, Imperial College London, London, UK; 4 Institute of Diagnostic Radiology and Neuroradiology, Greifswald University Hospital, Greifswald, Germany; 5 Institute for Medical Informatics, Biometry and Epidemiology, University Hospital Essen, Essen, Germany; 6 Department of Diagnostic and Interventional Radiology, University Hospital Augsburg, Augsburg, Germany; 7 Clinic for Diagnostic and Interventional Radiology, University Hospital Heidelberg, Heidelberg, Germany; 8 Institute of Social Medicine, Epidemiology and Health Economics, Charité – University Medicine Berlin, Berlin, Germany; 9 Institute of Clinical Epidemiology and Biometry, University of Würzburg, Würzburg, Germany; 10 State Institute of Health, Bavarian Health and Food Safety Authority, Erlangen, Germany; 11 Berlin Ultrahigh Field Facility (B.U.F.F.), Max-Delbrueck-Center for Molecular Medicine, Berlin, Germany; 12 Institute of Epidemiology, German Research Center for Environmental Health, Helmholtz Zentrum München, Neuherberg, Germany; 13 Institute for Medical Information Processing, Biometry and Epidemiology, Medical Faculty, Ludwig-Maximilians-Universität München, Munich, Germany; 14 German Diabetes Center (DZD E.V. - Partner Site Munich), Neuherberg, Germany; 15 Max-Delbrueck-Center for Molecular Medicine, Molecular Epidemiology Research Group, Berlin, Germany; 16 Max-Delbrueck-Center for Molecular Medicine in the Helmholtz Association (MDC), Biobank Technology Platform, Berlin, Germany; 17 Core Facility Biobank, Berlin Institute of Health at Charité - Universitätsmedizin Berlin, Berlin, Germany; 18 Charité - Universitätsmedizin Berlin, Corporate Member of Freie Universität Berlin and Humboldt-Universität Zu Berlin, Berlin, Germany; 19 Institute of Diagnostic and Interventional Radiology and Neuroradiology, University Hospital Essen, Essen, Germany; 20 Department of Molecular Epidemiology, German Institute of Human Nutrition, Nuthetal, Germany; 21 Institute of Nutritional Science, University of Potsdam, Nuthetal, Germany; 22 Institute for Community Medicine, Greifswald University Hospital, Greifswald, Germany; 23 Medical Image and Data Analysis Lab, Department of Radiology, University of Tübingen, Tübingen, Germany; 24 Department of Radiology, University of Freiburg, Freiburg, Germany; 25 Empirical Inference Department, Max-Planck Institute for Intelligent Systems, 72076 Tübingen, Germany; 26 Institute for AI and Informatics in Medicine, Klinikum Rechts Der Isar, Technical University of Munich, Munich, Germany

**Keywords:** Epidemiology, Magnetic resonance imaging, Whole body imaging

## Abstract

Large epidemiological studies such as the UK Biobank (UKBB) or German National Cohort (NAKO) provide unprecedented health-related data of the general population aiming to better understand determinants of health and disease. As part of these studies, Magnetic Resonance Imaging (MRI) is performed in a subset of participants allowing for phenotypical and functional characterization of different organ systems. Due to the large amount of imaging data, automated image analysis is required, which can be performed using deep learning methods, e. g. for automated organ segmentation. In this paper we describe a computational pipeline for automated segmentation of abdominal organs on MRI data from 20,000 participants of UKBB and NAKO and provide results of the quality control process. We found that approx. 90% of data sets showed no relevant segmentation errors while relevant errors occurred in a varying proportion of data sets depending on the organ of interest. Image-derived features based on automated organ segmentations showed relevant deviations of varying degree in the presence of segmentation errors. These results show that large-scale, deep learning-based abdominal organ segmentation on MRI data is feasible with overall high accuracy, but visual quality control remains an important step ensuring the validity of down-stream analyses in large epidemiological imaging studies.

## Introduction

Imaging data collected as part of large-scale epidemiological studies have the potential to provide unique insights into physiological and pathophysiological processes and determinants of health and disease in the general population. As part the UK Biobank Study (UKBB)^1^ and the German National Cohort (NAKO)^2^—two of the worldwide largest ongoing epidemiological studies with imaging data—comprehensive Magnetic Resonance Imaging (MRI) data are acquired to study anatomical and functional phenotypes of the entire body and of different organ systems including the central nervous system, the cardiovascular system, musculoskeletal system and the digestive system. In combination with a large amount of collected non-imaging parameters assessed through a plethora of standardized examinations in dedicated study centers, as well as results obtained from laboratory tests and linkage with secondary data sources, these data allow for comprehensive phenotyping of study participants. While most non-imaging data in these cohorts are easily accessible in the form of structured data formats, the extraction of useful phenotypic information from imaging data requires complex processing steps. Due to the overwhelming amount of data, image processing needs to be automated and tailored to specific scientific questions^3^.

Detection and segmentation of anatomical structures and organs is one of the most important steps of the image processing pipeline. Benefiting from substantial advances in deep learning techniques for medical image analysis, automated organ segmentation on MRI data has become feasible with satisfactory results in numerous applications^4^. Thus, deep learning-based organ segmentation has also become the method of choice for the analysis of large-scale imaging datasets. Several methodological studies have demonstrated the feasibility and performance of deep learning organ segmentation on UKBB and NAKO MRI data for different anatomic structures including the brain, the heart, the aorta, adipose tissue and abdominal organs^5–9^. While these studies report good overall results on small samples of larger cohorts with available ground truth, the deployment of automated organ segmentation on entire cohorts consisting of tens of thousands of participants without available ground truth is associated with substantial additional challenges regarding assessment of algorithm performance and quality assurance. Addressing these challenges is a prerequisite for the generation of valid scientific data for subsequent analyses and to avoid systematic errors or algorithm-induced bias, e. g. resulting from heterogeneous algorithm performance across sub-cohorts.

As of now, only few studies have addressed the deployment of automated organ segmentation on larger cohort study data and associated challenges^10,11^. In most population studies quality control of organ automated segmentations was either not performed at all or only algorithmic/automated segmentation quality ratings without any large-scale expert-based visual assessment was conducted. This limited approach to quality control is due to the massive effort and the limited availability of software tools that could accelerate this procedure. A recent study on automated organ segmentation using UKBB data employed automated fat–water swap detection and reported spot checks on few hundred subjects and obvious outliers^12^. To the best of our knowledge, only few studies in this context reported systematic visual quality control of image processing results by an expert in a major cohort study^13,14^.

Our contributions in this study are (1) the deployment of deep learning-based abdominal organ segmentation on 20,000 whole body MRI datasets from UKBB (1.5 Tesla MR) and NAKO (3 Tesla MRI), (2) large-scale visual expert quality control (QC) of these segmentations, (3) analysis of factors that impact segmentation quality ratings including epidemiological factors and image properties and (4) assessment of image-derived phenotypes based on segmentation quality.

## Results

**Figure 1:**
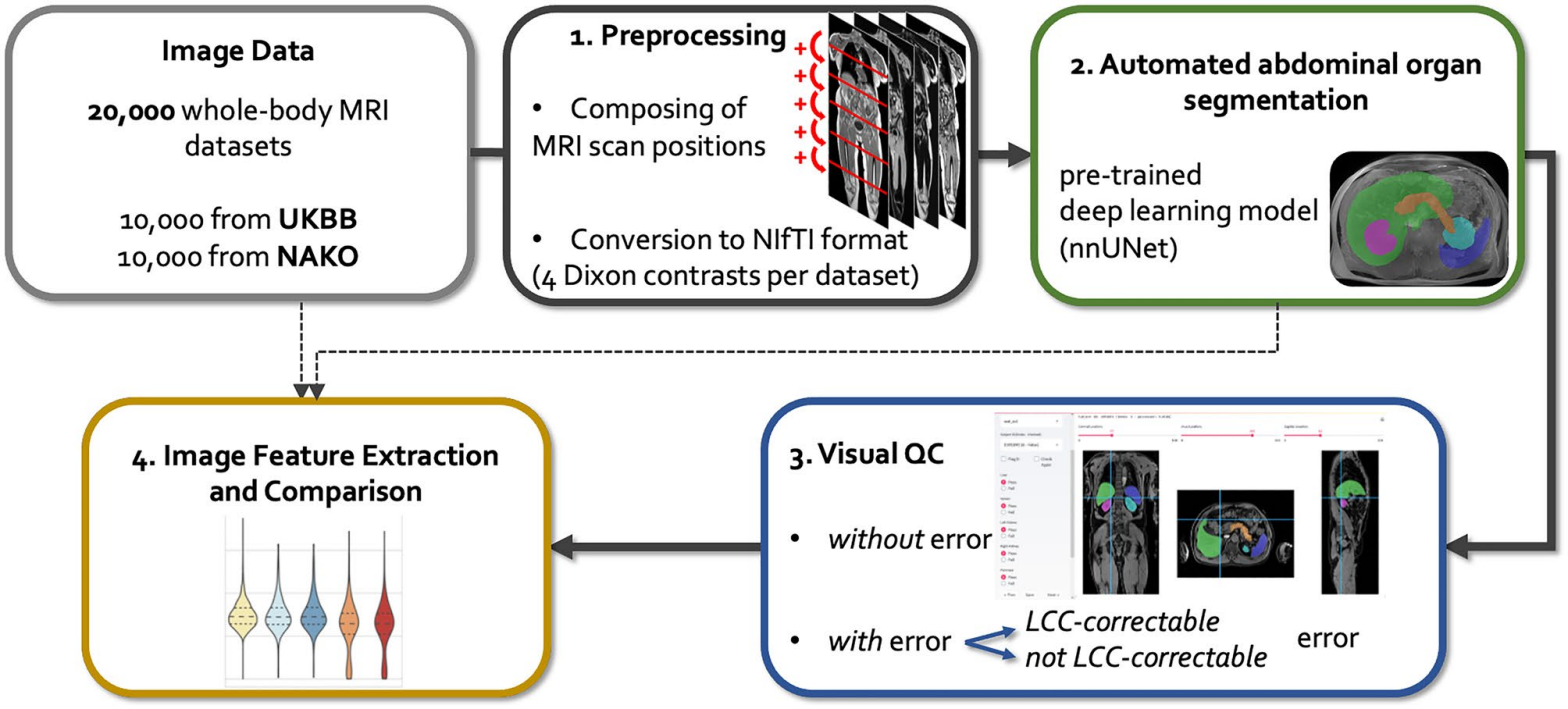
Summary of the data processing pipeline. In a first step (upper middle panel, black), image data from UKBB and NAKO were preprocessed by composing single MR acquisition stations and conversion to the NIfTI format. Subsequently (right upper panel, green) organs were automatically segmented on preprocessed image data. In a third step (lower right panel, blue), quality control of automated segmentations was performed visually and segmentation errors were categorized. Finally (lower left panel, yellow), shape features were extracted from image data based on organ segmentations. LCC = largest connected component.

A pictorial summary of the data processing pipeline consisting of preprocessing, automated organ segmentation, visual quality control and feature extraction is depicted in Fig. 1. A detailed description is provided in the Methods section.

### Cohort characteristics

The cohorts from UKBB and NAKO had comparable characteristics regarding sex distribution, body weight and body height (Table 1). Average age was markedly lower in the NAKO sub-cohort compared to the UKBB sub-cohort (51.9 vs. 63.1 years) due to different inclusion criteria as described in the methods section.

**Table 1:** Demographic characteristics of subjects.

	UKBB	NAKO
Number of subjects	10,000	10,000
Sex (F/M)	51.7% / 48.3%	49% / 51%
Age (SD) [years]	63.1 (7.5)	51.9 (11.4)
Weight (SD) [kg]	76.3 (15.2)	79.3 (16.4)
Height (SD) [cm]	169.1 (9.4)	171.7 (9.5)
BMI (SD) [kg/m^2^]	26.6 (4.4)	26.8 (4.8)

### Frequency of automated segmentation errors

During the visual QC process, we classified *errors* of automated organ segmentation into largest connected component (*LCC)- correctable errors* (i.e., the LCC of the segmentation mask corresponds to an error-free segmentation) and *not LCC*-*correctable errors* (see Methods section). This distinction is relevant as it directly relates to the possibility of automatically correcting errors through post processing by selecting the largest connected component of the segmentation mask in case of multiple components.

**Figure 2:**
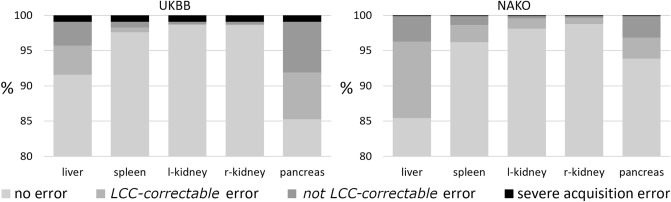
Frequency of segmentation error categories and severe acquisition errors (%) per organ for UKBB (left) and NAKO (right). LCC = largest connected component.

On a single-organ level most *errors* were observed for liver segmentation (8.4%/14.6% of datasets) and pancreas segmentation (14.8%, 6.1% of datasets) on UKBB / NAKO data respectively. When considering only *not LCC-correctable* errors, these percentages decreased to 3.6%/3.4% for liver segmentation and 7.2%/3.1% for pancreas segmentation on UKBB and NAKO data respectively (Fig. 2). Error rates were lowest for left and right kidney segmentation (below 2% in UKBB and NAKO, Fig. 2).

On a subject level, considering all organs, *no errors* or only *LCC-correctable* errors across all organs were observed in the majority of the data sets of both, UKBB and NAKO (92.3% / 88.5% respectively, Fig. 2). In other words, after appropriate error correction, approximately 9 in 10 datasets could be considered *error-free* in both, UKBB and NAKO.

### Association of automated segmentation errors with demographic and imaging factors

Statistical analysis revealed that the occurrence of composing artifacts between adjacent MRI acquisition blocks and the data source (UKBB vs. NAKO) were the main factors affecting the occurrence of segmentation errors. Composing artifacts were strongly associated with segmentation errors of liver and spleen (odds ratios of 136.5 and 18.8, respectively). UKBB data were markedly more likely to show *erroneous* pancreas segmentation while NAKO data sets were—to a lesser extent—more likely to show errors in segmentation of liver, spleen, and kidneys (Table 2).

**Table 2:** Association of epidemiological/imaging factors with segmentation errors (odds ratio (*p* values), statistically significant *p* values are in bold).

	Liver	Spleen	l-Kidney	r-Kidney	Pancreas
Age (SD: 11.16)	0.91 **(0.001)**	1.28 **(< 0.001)**	1.19 (0.027)	1.39 **(0.001)**	1.29 **(< 0.001)**
Sex (F: 0 / M: 1)	1.07 (0.21)	1.19 (0.07)	1.94 **(< 0.001)**	1.19 (0.29)	0.58 **(< 0.001)**
BMI (SD: 4.56)	1.21 **(< 0.001)**	1.29 **(< 0.001)**	1.71 **(< 0.001)**	1.35 **(< 0.001)**	0.84 **(< 0.001)**
Cohort (UKBB: 0 / NAKO: 1)	2.45 **(< 0.001)**	3.53 **(< 0.001)**	5.26 **(< 0.001)**	3.32 **(< 0.001)**	0.51 **(< 0.001)**
Composing Artifacts (False: 0 / True: 1)	136.52 **(< 0.001)**	18.82 **(< 0.001)**	2.91 **(< 0.001)**	4.86 **(< 0.001)**	1.02 (0.86)

Regarding the impact of epidemiological factors sex, age and BMI, we observed statistically significant effects to a far lesser extent compared to the impact of artifacts and data source. Notably, we observed a trend towards more frequent segmentation errors with increasing BMI for liver, spleen and kidneys.

### Impact of automated segmentation errors on image-derived features

To assess the impact of segmentation errors on potential downstream tasks we compared the distributions of shape features between organ segmentations *with* and *without* errors, in the latter category before and after error correction (by choosing the largest connected component of the segmentation mask).

**Figure 3:**
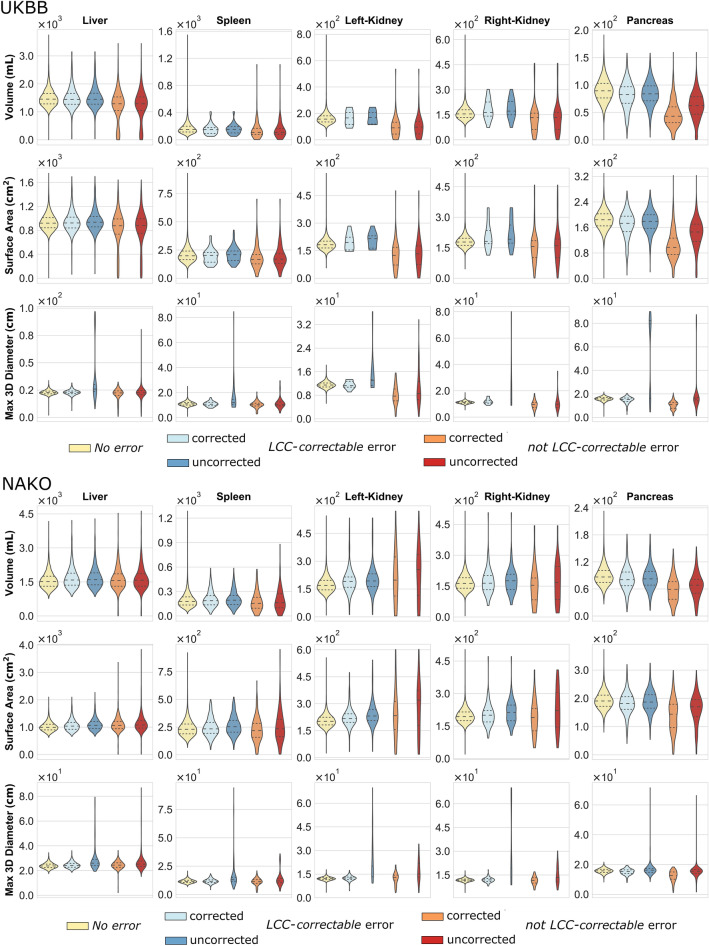
Impact of segmentation errors on extracted features (Top: UKBB, Bottom: NAKO). Comparison of segmentation mask categories (from left to right within each subplot: *without error* (yellow), corrected *largest connected component* (*LCC)*-*correctable* error (dark blue), uncorrected *LCC*-*correctable* error (light blue), corrected *not LCC*-*correctable* error (dark red), uncorrected *not LCC*-*correctable* error (light red) across organs (subplots from left to right: liver, spleen, left kidney, right kidney, pancreas) and across shape features (subplots from top to bottom: volume, surface area, maximum 3D diameter).

Overall, we observed that shape features drawn from liver segmentations were least affected by the presence of errors while the effect of errors was more pronounced for kidneys and pancreas segmentations (Fig. 3).

For the features “volume” and “surface area”, only slight feature deviations compared to the *error-free* case were observed on data with *LCC-correctable* errors while pronounced deviations were observed on data with *not LCC-correctable* errors. For illustration, the mean pancreatic volume on *error-free* segmentations was 90.2 ml/88.8 ml on UKBB/NAKO respectively and 84.4 ml/84.5 ml on data with *LCC-correctable* errors and 62 ml/66.8 ml on data with *not LCC-correctable* errors (Fig. 3).

LCC error correction (by choosing the largest connected component of the segmentation mask) had only minor effects on the shape features “volume” and “surface area” for *LCC-correctable* errors—implying that incorrect connected segmentation components were of small quantitative significance in this regard. In contrast, performing LCC correction on *not LCC-correctable* segmentation error resulted in a further deviation of shape features compared to *error-free* data—implying that LCC correction is even harmful in these error cases. For illustration, after LCC error correction, the mean pancreatic volume was 81.3 ml/82.5 ml on data with *LCC correctable* errors and 47.1 ml/58.5 ml on data with *not LCC-correctable* errors (for UKBB and NAKO respectively).

The feature “maximum 3D diameter” was markedly more susceptible to the presence of errors and showed the highest deviation between *error-free* and *erroneous* segmentation masks in both data sets (Fig. 3). For this feature, error correction resulted in alignment of feature distributions to the *error-free* case in case of *LCC-correctable* errors and to a lesser extent for *not LCC-correctable* errors (Fig. 3).

## Discussion

In this study, we applied a deep learning-based organ segmentation model to 20,000 whole body MRI datasets from the UKBB and NAKO cohorts and performed standardized visual quality analysis of segmentation results.

We found that overall segmentation accuracy was high in both cohorts, at 1.5 and 3 Tesla, while segmentation errors were observed in a small but non-negligible proportion of datasets. When considering only *not LCC-correctable* errors, these occurred in only a small fraction of datasets in both cohorts: After correction of *LCC-correctable* errors about 90% of all datasets were error-free for all organs on UKBB (88.5%) and NAKO (92.3%) data.

Detailed analysis revealed that liver and pancreas were most susceptible to segmentation errors. The occurrence of composing artifacts was mainly associated with liver segmentation errors and pancreas segmentation errors occurred more frequently in the UKBB cohort. The effect of composing artifacts on liver segmentation can be explained by the anatomic position of the liver below the diaphragm and its longitudinal extent which can results in relative displacement of adjacent MR acquisition stations in different respiratory states. The higher frequency of pancreas segmentation errors on UKBB data is probably partly a result of the lower native image resolution in the UKBB imaging protocol. A slight observed trend for increasing artifact frequency with higher BMI is not clearly explainable as one might expect an easier segmentation task with increasing adipose tissue content surrounding organs. However, other factors, most notably relative underrepresentation of subjects with high BMI in the training data may potentially explain this result.

Importantly, we observed that the occurrence of segmentation errors resulted on marked distribution shift of shape features extracted from organ segmentations. This effect was most pronounced for kidneys and pancreas. This result suggests that thorough quality control of automated segmentation results is necessary when it comes to ensuring the validity of downstream analysis.

Interestingly, feature distributions of segmentations with *LCC-correctable* errors were noticeably closer to the error-free category compared to segmentations with *not LCC-correctable* errors. This implies that *LCC-correctable* errors are also less severe compared to *not LCC-correctable* errors. Also, as to be expected, automated error correction by choosing the largest connected component of segmentation masks was only effective for *LCC-correctable* errors. This result underlines that automated error correction through post-processing still requires steps of visual quality control.

Our results are in line with previous studies reporting higher frequencies of segmentation errors especially of the pancreas compared to other major abdominal organs^5^. Previous studies reported the small organ size and complex anatomical background as the main factors making pancreas segmentation challenging. Thus, in accordance with our observation, higher image resolution is beneficial for accurate pancreas segmentation. In the context of UKBB, the use of an additional, high resolution MR acquisition focused on the pancreas has been reported to improve accuracy of automated pancreas segmentation^15^.

In this study, quality analysis was performed visually by an experienced imaging expert; smaller segmentation errors along organ borders may have easily be missed. It is conceivable that methodological developments may allow for more accurate, automated quality analysis of organ segmentation in the future. First studies have shown the feasibility of automated quality control in UKBB, though they are hardly a replacement for visual quality analysis as the scope of their assessment remains limited^6,16^. We aim to explore this avenue with further methods, e.g. using model uncertainty as a surrogate for segmentation quality, in future studies. As a future strategy, it may additionally be useful to focus on the occurrence of specific image artifacts, such as composing artifacts, to pre-filter data sets with that require additional visual inspection.

The results of this study show that—despite high overall accuracy of deep learning organ segmentation—a substantial number of automated segmentation errors can occur that cannot be eliminated by simple post processing. Particularly in the context of epidemiological studies—where segmentation results will likely be used in multiple follow-up studies by different research groups—this demands visual quality control of all segmentation masks. Methodological improvements regarding robustness of deep learning methods may lessen this necessity in the future.

We conclude that large-scale, deep learning-based automated abdominal organ segmentation on MRI data is feasible with overall high accuracy, but visual quality control remains an important step ensuring the validity of down-stream analyses in large epidemiological imaging studies.

## Materials and methods

### Image data

Data in this study were acquired from the UK Biobank study (UKBB) in the United Kingdom and from the German National Cohort study (NAKO) in Germany, which obtained written informed consents from all subjects and approved our data analysis. Research involving human participants was performed in accordance with the Declaration of Helsinki in both studies. The analysis of anonymized data from these studies was approved by the ethics committee of the Medical Faculty of the University in Tübingen.

Cohort characteristics and imaging protocols differ between these two large-scale population studies. The UKBB study aims to image 100,000 healthy UK participants between 40 and 69 years of ages; MRI are performed on 1.5 T clinical MRI scanners (Magnetom Aera, Siemens Healthineers, Erlangen, Germany). The NAKO study enrolled 30,000 participants between 20 and 69 years of age from the general German population for the MRI part of the study; 3 T clinical MRI scanners (Magnetom Skyra, Siemens Healthineers, Erlangen, Germany) are used in the NAKO.

In both studies, as part of an extensive imaging protocol, whole-body T1-weighted dual echo gradient echo (GRE) sequences are acquired with the following parameters (UKBB: pixel size [mm^2^], 2.23 × 2.23; slice thickness [mm], 3–4.5; echo times [ms], 2.39 / 4.77; repetition time [ms], 6.69; flip angle [°], 10; NAKO: pixel size [mm^2^], 1.2 × 1.2; slice thickness [mm], 3; echo times [ms], 1.23 / 2.46; repetition time [ms], 4.36; flip angle [°], 9). Further protocol details have been reported in^2^ and^17^. Based on these sequences, four MRI contrasts (Dixon contrasts), namely water, fat, in-phase (IP) and out-of-phase (OP) are made available in both studies. A total of 20,000 participants (10,000 subjects from each study) were available in our analysis. Notably, spatial resolution is markedly higher in NAKO, mainly due to the higher magnetic field strength of deployed MRI scanners.

### Data pre-processing and automated organ segmentation

The source data for this study consisted of whole-body T1-weighted images from 6 acquisition stations for the UKBB (neck-to-knee) and 4 acquisition stations for the NAKO (neck to upper thigh) stored in the DICOM format. Following the approach described in^5^ these images were converted into four 3D image files per subject, each of which corresponds to one of the four Dixon contrast, and stored in the NIfTI format. As part of this process, a composing step stitching together the single MRI acquisition blocks was performed on UKBB data using publicly available in-house software (https://github.com/biomedia-mira/stitching)^18^, whereas NAKO data were available as pre-composed whole-body data sets. For automated segmentation of the liver, the spleen, the left and right kidneys as well as the pancreas, publicly available, pre-trained deep learning-based models for abdominal organ segmentation described in^5^ were used for this study (code: https://github.com/BioMedIA/UKBB-GNC-Abdominal-Segmentation, trained models: https://gitlab.com/turkaykart/ukbb-gnc-abdominal-segmentation). These models, based on a standardized U-Net architecture (nnU-Net) detailed in^4^, were previously trained on UKBB and the NAKO training data based on 400 manually labeled image volumes and were extensively validated in a previous study^5^. All four Dixon image contrasts were given as model inputs. The UKBB model was deployed on a GPU workstation equipped with 2 Titan RTX GPUs (NVIDIA, Santa Clara, USA) whereas the NAKO model was deployed on a dedicated GPU server using two Tesla V100 GPUs (NVIDIA, Santa Clara, USA).

The first 10,000 complete data sets on which the data processing chain was technically successful (i.e. loading and algorithmic processing of data was computationally possible) were drawn from each, the UKBB and NAKO data pool for this study.

### Visual quality control (QC)

**Figure 4:**
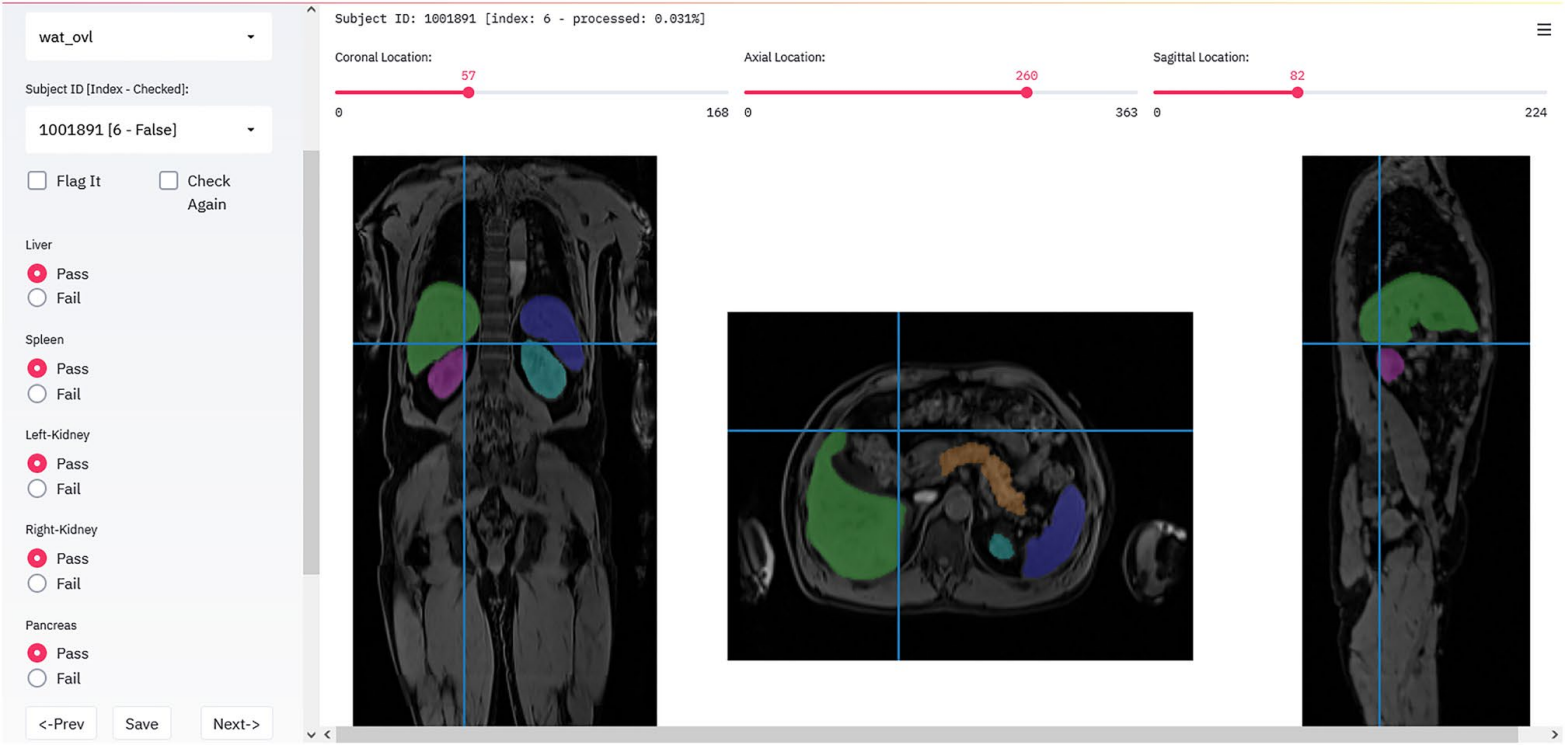
Quality analysis tool (SegQC). It enables visual assessment of segmentation quality by an expert in an efficient and scalable setup, specifically designed for population imaging studies with flexible caching and indexing to simplify the QC process.

For visual quality control, a QC tool with an interactive graphical user interface (GUI), called SegQC, was developed in Python (code available at: https://github.com/BioMedIA/UKBB-GNC-QC-Tool). It enables visual assessment of segmentation quality by an expert in an efficient and scalable setup, specifically designed for population imaging studies with flexible caching and indexing to simplify the QC process (Fig. 4). Similar assistive tools aiming for expediating assessments and reproducibility have already shown the benefits of such interactive quality control in neuroimaging^19–21^.

SegQC’s graphical user interface was created with an open-source application programming interface (API) Streamlit (https://github.com/streamlit/streamlit), which enables the development of a web app accessible through a web browser. In addition to working with large datasets, SegQC offers various features such as different viewing orientations (coronal, sagittal and axial), overlay of segmentation masks and generation of maximum intensity projections (MIP) as well as storage of quality ratings as a csv file. The tool allows one to assess organ segmentation quality slice by slice in different views and/or visualize different organ segmentations all in one screen using MIPs. In addition, the user can adjust the granularity of QC rating options through a configuration file. Furthermore, one can navigate subjects consecutively, select subjects based on IDs as well as flag interesting subjects for later re-assessment.

Using SegQC, visual segmentation quality analysis of all 20,000 data sets was performed by an expert radiologist (SG—11 years of experience). The overall aim of this process was to identify data sets with relevant segmentation errors and to assess to which extent these errors were correctable.

**Figure 5:**
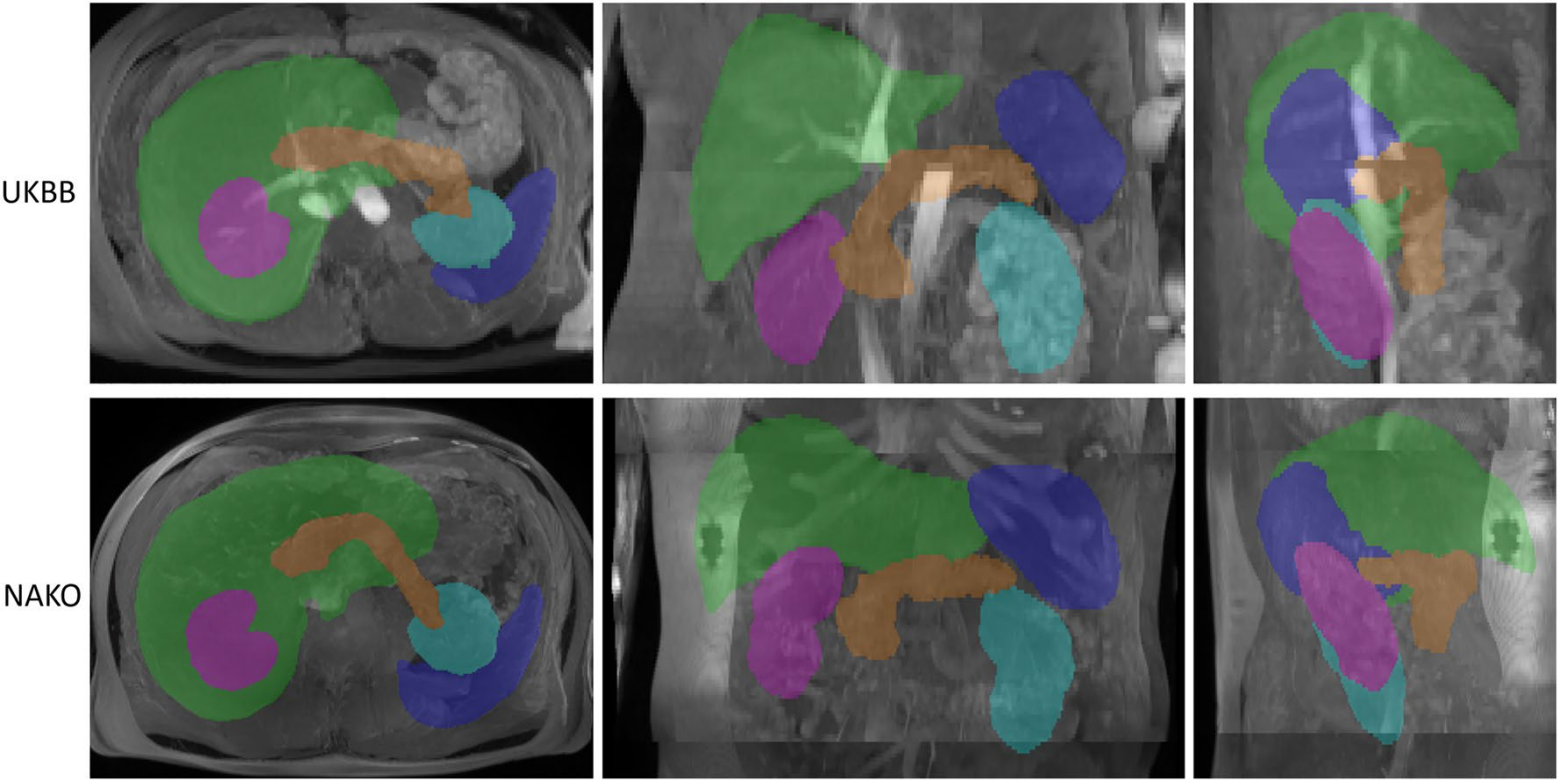
Axial (left), coronal (middle) and sagittal (right) maximum intensity projections of error-free automated segmentation results (green = liver, blue = spleen, turquoise = left kidney, pink = right kidney, orange = pancreas) of UKBB (top) and NAKO (bottom).

Organ segmentations were defined as *“without error” (“error-free”, “no error”)* in case no segmentation error was visually perceivable on coronal, axial or sagittal MIP images and segmentation masks consisted of a single connected component (examples given in Fig. 5).

**Figure 6:**
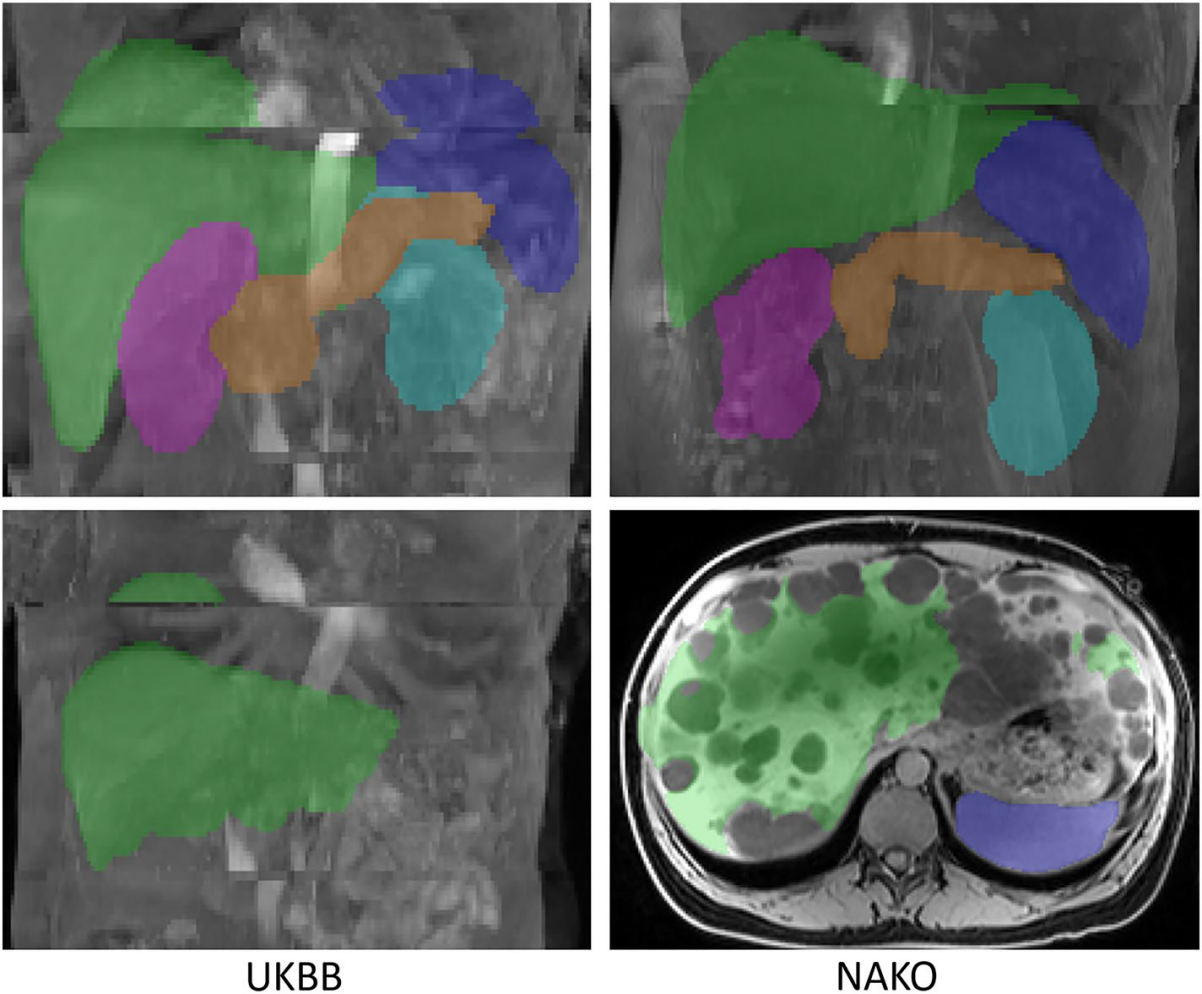
Examples for errors (left: UKBB, right: NAKO). Top: Example images of *not largest connected component (LCC)*-*correctable* segmentation errors caused by respiratory misalignment and resulting composing artifact of the liver and spleen (left) and the liver (right). Bottom: *LCC*-*correctable* segmentation error of the liver due to a composing artifact (left) and *not LCC-correctable* error of the liver due to cystic liver disease (right).

Accordingly, automated segmentations for each organ were defined as *“erroneous”* or “*with error*” if segmentation errors were visually perceivable on coronal, axial or sagittal MIP images (as only partly segmented organ or as segmentation mask exceeding organ boundaries) or if the segmentation mask consisted of more than one connected component for a single organ (Fig. 6).

Relevant segmentation errors due to multiple connected components were categorized as “*largest-connected-component (LCC)-correctable”* in case the largest connected component (LCC) of the segmentation mask corresponded to the target organ and showed no relevant error (Fig. 6). By this definition, an *LCC-correctable erroneous* organ segmentation can be corrected by discarding smaller connected components and retaining the largest. *Erroneous* segmentations that were not *LCC-correctable* were categorized as *not LCC-correctable*.

In addition, the existence of composing artifacts between adjacent MRI acquisition stations was visually assessed and recorded. As whole-body MRI data were acquired in several acquisition blocks with subsequent composing to a single 3D image block (see above), spatial inconsistencies between adjacent blocks can cause image artifacts. Composing artifacts were defined as inconsistencies between two adjacent MRI acquisition stations that resulted in missing or duplicated anatomical structures (e.g., due to different respiratory states along the diaphragm). In case target organs were partially missing or duplicated due to composing artifacts, segmentations were considered erroneous even if they technically correctly corresponded to the respective organs since these segmentations did not correctly describe the actual organ anatomy (Fig. 6).

In a small number of datasets (92/10,000 UKBB data sets and 12/10,000 NAKO data sets), severe image acquisition errors were observed (such as fat/water swaps and MR signal alterations) resulting in severely altered image properties and relevant segmentation errors in all organs. These data sets were discarded from further statistical analysis.

### Segmentation error correction

As described above, by definition, an *LCC-correctable* error occurred in case automated organ segmentation masks consisted of multiple connected components and the largest of these components corresponded to an *error-free* segmentation of the target organ. For such organ segmentations with *LCC-correctable* errors correction was possible by choosing only the largest connected component (LCC correction), generating the final segmentation map. This ensured that LCC-corrected segmentation masks of organs with *LCC-correctable* errors did not have relevant errors in contrast to simply applying this post-processing step without previous qualitative visual assessment. For *not LCC-correctable* errors this is in general not the case. In order to investigate to what extent blind application of LCC correction (without prior identification of *not LCC-correctable* errors) can lead to quantification errors, we investigated the effect of LCC correction on organ features without and with LCC correction.

### Extraction of organ phenotypes

Three image-derived features were extracted from the composed images and their corresponding segmentation maps per organ and subject: organ volume, organ surface area and maximum 3D organ diameter. Feature extraction was implemented in Python using the PyRadiomics^22^ package.

### Statistical analysis of QC results

Statistical analysis consisted of several stages. We first extracted the epidemiological parameters, namely age, sex, weight and height from the UKBB and NAKO meta-data for all 20,000 subjects. For the UKBB cohort, age was calculated from the MRI examination date (data field ID 53), year of birth (data field ID 34) and month of birth (data field ID 52). Sex, weight, and height were acquired from the data field IDs 31, 21,002 (if missing 12,143), 50 (if missing 12,144), respectively. For the NAKO cohort, age was drawn from the field df100_age, sex was drawn from the field df100_sex, size from the field anthro_groe, anthro_groe_eigen or anthro_groe_man (in this order depending on data availability) and weight from anthro_gew, anthro_gew_eigen or anthro_gew_man (in this order depending on data availability).

For further analysis, we first calculated the frequency of segmentation errors per participant and organ for each cohort. To identify associations between segmentation quality ratings and epidemiological and imaging factors, multivariable logistic regression was performed. Regression analysis was performed for each organ individually. In this analysis, the binary dependent variable was the presence of segmentation errors (yes / no) per organ and independent variables were age, sex, BMI, data source (UKBB / NAKO) and the presence of composing artifacts (yes / no). With multivariable logistic regression, we calculated odd ratios per organ error category and associated *p* values. The odd ratios for continuous variables (age and BMI) were scaled using the standard deviation (SD) to reflect the odds per SD change in that variable. *p* Values < 0.01 were considered statistically significant, accounting for multiple (five-fold) testing.

In addition, we compared the distributions of extracted shape features (volume, surface area, maximum 3D diameter) of segmentation masks *with* and *without* errors. In the latter case we compared feature distributions before and after LCC correction (by choosing the largest connected component).

Statistical analyses were performed using the Statsmodel library^23^ in Python.

## Data Availability

The data that support the findings of this study were provided from UK Biobank (www.ukbiobank.ac.uk) and NAKO (www.nako.de) but restrictions apply to the availability of these data, which were used under license for the current study, and so are not publicly available. Applications for NAKO data can be directed to the transfer unit at https://transfer.nako.de/.
